# Analysis of human visual experience data

**DOI:** 10.1167/jov.26.7.1

**Published:** 2026-07-01

**Authors:** Johannes Zauner, Aaron Nicholls, Lisa A. Ostrin, Manuel Spitschan

**Affiliations:** 1 Technical University of Munich, TUM School of Medicine and Health, Department Health and Sport Sciences, Chronobiology & Health, Munich, Germany; 2 Translational Sensory & Circadian Neuroscience, Max Planck Institute for Biological Cybernetics, Tübingen, Germany; 3 Reality Labs Research, Redmond, WA, USA; 4 University of Houston College of Optometry, Houston, TX, USA; 5 TUM Institute for Advanced Study (TUM-IAS), Technical University of Munich, Garching, Germany

**Keywords:** wearable, light logging, viewing distance, near-work, visual experience, metrics, circadian, myopia, risk factors, time series, spectral analysis, spatial analysis, open-source, reproducibility

## Abstract

Exposure to the optical environment—often referred to as *visual experience*—profoundly influences human physiology and behavior across multiple time scales. In controlled laboratory settings, stimuli can be held constant or manipulated parametrically. However, such exposures rarely replicate real-world conditions, which are inherently complex and dynamic, generating high-dimensional datasets that demand rigorous and flexible analysis strategies. This tutorial presents an analysis pipeline for visual experience datasets, with a focus on reproducible workflows for human chronobiology and myopia research. Light exposure and its retinal encoding affect human physiology and behavior across multiple time scales. Here we provide step-by-step instructions for importing, visualizing, and processing viewing distance and light exposure data. This includes time-series analyses for working distance, biologically relevant light metrics, and spectral characteristics. The tasks are standardized through the open-source R package LightLogR. By leveraging a modular approach, the tutorial supports researchers in building flexible and robust pipelines that accommodate diverse experimental paradigms and measurement systems.

## Introduction

Exposure to the optical environment—often referred to as *visual experience*—profoundly influences human physiology and behavior across multiple time scales. Two notable examples, from distinct research domains, can be understood through a common retinally referenced framework.

The first example relates to the nonvisual effects of light on human circadian and neuroendocrine physiology. The light–dark cycle entrains the circadian clock, and light exposure at night suppresses melatonin production (Blume, Garbazza, & Spitschan, 2019; Brown et al., 2022). The second example concerns the influence of visual experience on ocular development, particularly myopia. Time spent outdoors—which features distinct optical environments—has been consistently associated with protective effects on ocular growth and health outcomes (Dahlmann-Noor, Bokre, Khazova, & Price, 2025).

In controlled laboratory settings, light exposure can be held constant or manipulated parametrically. In contrast, real-world conditions are inherently complex and dynamic and cannot be captured by single spot measurements. As people move in and between spaces (indoors and outdoors) and move their body, head, and eyes, exposure to the optical environment varies significantly (Webler, Spitschan, Foster, Andersen, & Peirson, 2019) and is modulated by behavior (Biller, Balakrishnan, & Spitschan, 2024). Wearable devices for measuring light exposure have thus emerged as vital tools to capture the ecological visual experience. These tools generate high-dimensional datasets that demand rigorous and flexible analysis strategies.

Starting in the 1980s (Okudaira, Kripke, & Webster, 1983), technology to measure optical exposure has matured, with miniaturized illuminance sensors now (in 2026) very common in consumer wearables (van Duijnhoven et al., 2025). In research, several devices are available that differ in functionality, ranging from small pins measuring ambient illuminance (Mohamed et al., 2021) to head-mounted multimodal devices capturing nearly all relevant aspects of visual experience (Gibaldi, Harb, Wildsoet, & Banks, 2024). Increased capabilities in wearables bring complex, dense datasets. These go hand-in-hand with a proliferation of metrics, as highlighted by recent review papers in both circadian and myopia research (Hartmeyer & Andersen, 2023; Hönekopp & Weigelt, 2023).

At present, the analysis processes to derive metrics are often implemented on a per-laboratory or even per-researcher basis. This fragmentation is a potential source of errors and inconsistencies between studies and consumes considerable researcher time (Hartmeyer, Webler, & Andersen, 2022), and these bespoke processes and formats hinder harmonization or meta-analysis across multiple studies. It is very common that more time is spent preparing data than gaining insights through rigorous statistical analysis. These preparation tasks are best handled or at least facilitated by standardized, transparent, community-based analysis pipelines (Zauner, Udovicic, & Spitschan, 2024).

In circadian research, the R package LightLogR was developed to address this need (Zauner, Hartmeyer, & Spitschan, 2025). LightLogR is an open-source, MIT-licensed, community-driven package specifically designed for data from wearable light loggers and optical radiation dosimeters. It contains functions to calculate over 60 different metrics used in the field (Hartmeyer & Andersen, 2023). The package functions come with light-related defaults, but they remain fundamentally agnostic to modality. As a result, parameters such as viewing distance and light spectra, both highly relevant to myopia research (Hönekopp & Weigelt, 2023), can easily be handled.

In this article, we demonstrate that LightLogR's analysis pipelines and metric functions apply broadly across the field of visual experience research, not just to circadian rhythms and chronobiology. Our approach is modular and extensible, allowing researchers to adapt it to a variety of devices and research questions. Emphasis is placed on clarity, transparency, and reproducibility, aligning with best practices in scientific computing and open science. We use example data from two devices (worn by different individuals and at different times) to showcase the LightLogR workflow with metrics relevant to myopia research, covering working distance, (day)light exposure, and spectral analysis. Readers are encouraged to re-create the analysis using the provided code. All necessary data and code are openly available in the GitHub repository (https://github.com/tscnlab/ZaunerEtAl_JVis_2026/).

**Scope**

This article focuses on workflows for deriving condensed metrics from time-series data collected with wearable devices in the visual experience domain. Specifically, we address illuminance, viewing distance, and spectral irradiance. Example datasets from two types of wearable devices are used for illustration.

Many relevant considerations arise when collecting data with wearable devices. This article covers only a subset of these. In particular, it does not address the following:

• Device selection (see, e.g., van Duijnhoven et al., 2025; Zauner, Baraas et al., 2025; Zauner, Stefani, Biller, Guidolin, & Spitschan, 2025)
• Measurement accuracy or device calibration
• Auxiliary data such as sleep/wake information (see, e.g., Guidolin, Zauner, Hartmeyer, & Spitschan, 2026; Zauner et al., 2026)

More information on those aspects can be found in the Technical Guide for Wearable Optical Radiation Dosimetry and Visual Experience Assessment (Zauner, Baraas et al., 2025).

To demonstrate the workflows, this article uses expert-informed definitions of metrics and metric parameters (see, e.g., Tables 1, 2, and the nonwear detection rules based on activity data described in Supplement 1). These definitions and thresholds should not be interpreted as universal standards, nor are they hard-coded into the software package. For any application, parameter choices must be tailored to the research domain, study context and design, and the specifications of the wearable device. The demonstrated software supports this expressly.

Further, the article is split up into the main analysis part, where all metrics are calculated, and Supplement 1, where data are imported, screened, and prepared. Thus, the reader is referred to Supplement 1 for all aspects regarding data formats, preparation steps, and handling of gaps (i.e., missing data).

Lastly, the example data used in the article do not stem from a controlled experimental data collection but consist of data gathered in an ecological setting without a fixed protocol. Given the substantial interindividual differences in visual experience metrics, and because the analyses focus on one participant at a time, the reported results should be interpreted as illustrative rather than representative of typical or population-level values.

**Table 1.** Overview of metrics. In all cases, the averages for weekday, weekend, and the mean daily value are calculated. Definitions are shown in Table 2.

No.	Name	Implementation^a^
**Distance**		
1	Total wear time daily	durations()*
2	Duration of Near work, Intermediate work, Near + Intermediate work, or per each *Distance range* (10-cm steps)	filter for distance range + durations()* (for single ranges) or grouping by distance range + durations()* (for all ranges)
3	Frequency of Continuous near work	extract_clusters()* + summarize_numeric()*
4	Frequency, duration, and distances of *Near* *work episodes*	extract_clusters()* + extract_metric()* + summarize_numeric()*
5	Frequency and duration of *Visual breaks*	extract_clusters()* + filter
**Light**		
6	Light exposure (in lux)	summarize_numeric()*
7	Duration per *Outdoor range*	grouping by Outdoor range + durations()*
8	The number of times light level changes from indoor (<1,000 lx) to outdoor (>1,000 lx)	extract_states()* + summarize_numeric()*
9	Longest period above 1,000 lx	period_above_threshold()*
**Spectrum**		
10	Ratio of short- vs. long-wavelength light	spectral_integration()* + summarize_numeric()*
11	Melanopic daylight efficacy ratio (MDER)	spectral_integration()* + summarize_numeric()*
12	Short-wavelength light at certain times of day	spectral_integration()* + filter_Time()* (for defined times) or cut_Datetime()* (for regular time intervals) or add_photoperiod()* (for solar times) + grouping by time state + summarize_numeric()*

a Functions from LightLogR are presented with an asterisk. Nonasterisk functions refer to tidyverse functions.

**Table 2.** Definitions of terms and metrics.

Term/metric	Description/pseudo formula
Total wear time	Σ(t)*dt, where t: valid observations
Mean daily	5* wee k¯ day +2* wee k¯ end 7
Near work	working distance, [15, 60) cm
Intermediate work	working distance, [60, 100) cm
Total work^a^	working distance, [10, 120) cm
Distance range	working distance,
	[15, 20) cm, Extremely near
	[20, 30) cm, Very near
	[30, 40) cm, Fairly near
	[40, 50) cm, Near
	[50, 60) cm, Moderately near
	[60, 70) cm, Near intermediate
	[70, 80) cm, Intermediate
	[80, 90) cm, Moderately intermediate
	[90, 100) cm, Far intermediate
Continuous near work	$T_{t}=\Sigma \left(t\right)*dt,\;\mathrm{where}$ working distance, [20, 60) cm, *T*_t_ ≥ 15 minutes, *T*_interruptions_ ≤ 30 seconds
Near-work episodes	$T_{t}=\Sigma \left(t\right)*dt,\;\mathrm{where}$ working distance, [20, 60) cm, *T*_interruptions_ ≤ 20 seconds
Ratio of daily near work	T near work T total wear
Visual break	$T_{t}=\Sigma \left(t\right)*dt,\;\mathrm{where}$ working distance ≥ 100 cm, *T*_t_ ≥ 20 seconds,
	*T*_*t* − 1 (working distance < 100 cm)_ ≤ 20 minutes
Outdoor range	illuminance, [1000, 2000) lx, Outdoor bright [2000, 3000) lx, Outdoor very bright [3000, ∞) lx, Outdoor extremely bright
Light exposure^b^	$Illum\bar{i}nance$
Spectral bands	*S*pectral irradiance, [400, 500] nm, short − wavelength light [600, 700] nm, long − wavelength light
Ratio of short- vs. long-wavelength light	Ee, short wavelength Ee, long wavelength

a The upper threshold refers to the Clouclip's maximum distance measurement and is not theoretically based. ^b^This deviates from the common definition of luminous exposure, which is the sum of illuminance measurements scaled to hourly observation intervals.

## Method and materials

### Software

This tutorial was built with Quarto (https://quarto.org), an open-source scientific and technical publishing system that integrates text, code, and code output into a single document. The source code to reproduce all results is included and accessible via the Quarto document's code tool menu. All analyses were conducted in R (Version 4.5.0, “How About a Twenty-Six”) using LightLogR (Version 0.10.0 “High noon”). We also used the tidyverse suite (Version 2.0.0) for data manipulation (which LightLogR follows in its design) and the gt package (Version 1.1.0) for generating summary tables. A comprehensive overview of the R computing environment is provided in the session info (see Session info section of the online tutorial, https://tscnlab.github.io/ZaunerEtAl_JVis_2026/).

### Metric selection and definitions

In March 2025, two workshops with myopia researchers—initiated by the Research Data Alliance (RDA) Working Group on Optical Radiation Exposure and Visual Experience Data—focused on current needs and future opportunities in data analysis, including the development and standardization of metrics. Based on expert input from these workshops, the authors of this tutorial compiled a list of visual experience metrics, shown in Table 1. These include many currently used metrics and definitions (Bhandari & Ostrin, 2020; Wen et al., 2019; Wen et al., 2020; Williams, Bakshi, Ostrin, & Ostrin, 2019), as well as new metrics enabled by spectrally resolved measurements. While they are not derived from a formal consensus process, they are expert-informed and used in current scientific research and thus will serve as example definitions for metrics and thresholds throughout this article.

Table 2 provides definitions for the terms used in Table 1. Note that specific definitions may vary depending on the research question or device capabilities.

It should be noted that although daylight levels can far exceed the thresholds defined in Table 2—and may reach or even exceed 10^5^ lux—empirical daylight levels measured at eye level are much lower, typically around 10^3^ lux, especially when considering aggregated time-series data over minutes to hours.

### Devices

Data from two wearable devices are used in this analysis:

• Clouclip: A wearable device that measures viewing distance and ambient light simultaneously (Glasson Technology Co., Ltd, Hangzhou, China; Bhandari & Ostrin, 2020; Wen et al., 2020; Wen et al., 2021). The Clouclip provides a simple data output with only distance (working distance, in centimeters) and illuminance (ambient light, in lux). Data in our example were recorded at 5-second intervals. Approximately 1 week of data (∼120,960 observations) is about 2 to 4 MB in size. For the Clouclip device, the dataset was obtained in November 2020 from a 30-year-old male graduate student in Texas, United States, and was previously presented as part of a published study (Bhandari, Mirhajianmoghadam & Ostrin, 2021). The light exposure profile reflects relatively limited time outdoors, which is consistent with patterns reported in individuals engaged in intensive academic training and the pandemic during 2020.
• Visual Environment Evaluation Tool (VEET): A head-mounted multimodal device that logs multiple data streams (Reality Labs Research, Menlo Park, CA, USA; Sah, Narra, & Ostrin, 2025; Sullivan et al., 2024). The VEET dataset used here contains simultaneous measurements of distance (via a time-of-flight sensor), ambient light (illuminance), activity (accelerometer and gyroscope), and spectral irradiance (multichannel light sensor). Data were recorded at 2-second intervals, yielding a very dense dataset (∼270 MB per week). For the VEET device, the dataset comes from pilot data collection of a researcher in Texas, United States, under naturalistic conditions in June 2024 (light and distance) and June 2025 (spectral data). The researcher was working in a typical office building with limited daylight availability during office hours and limited time outdoors overall.

The distribution of light exposure in both datasets is typical for indoor and partial outdoor times and is discussed further at the beginning of the Results section for light exposure.

### Data-processing summary

**Figure 1.**
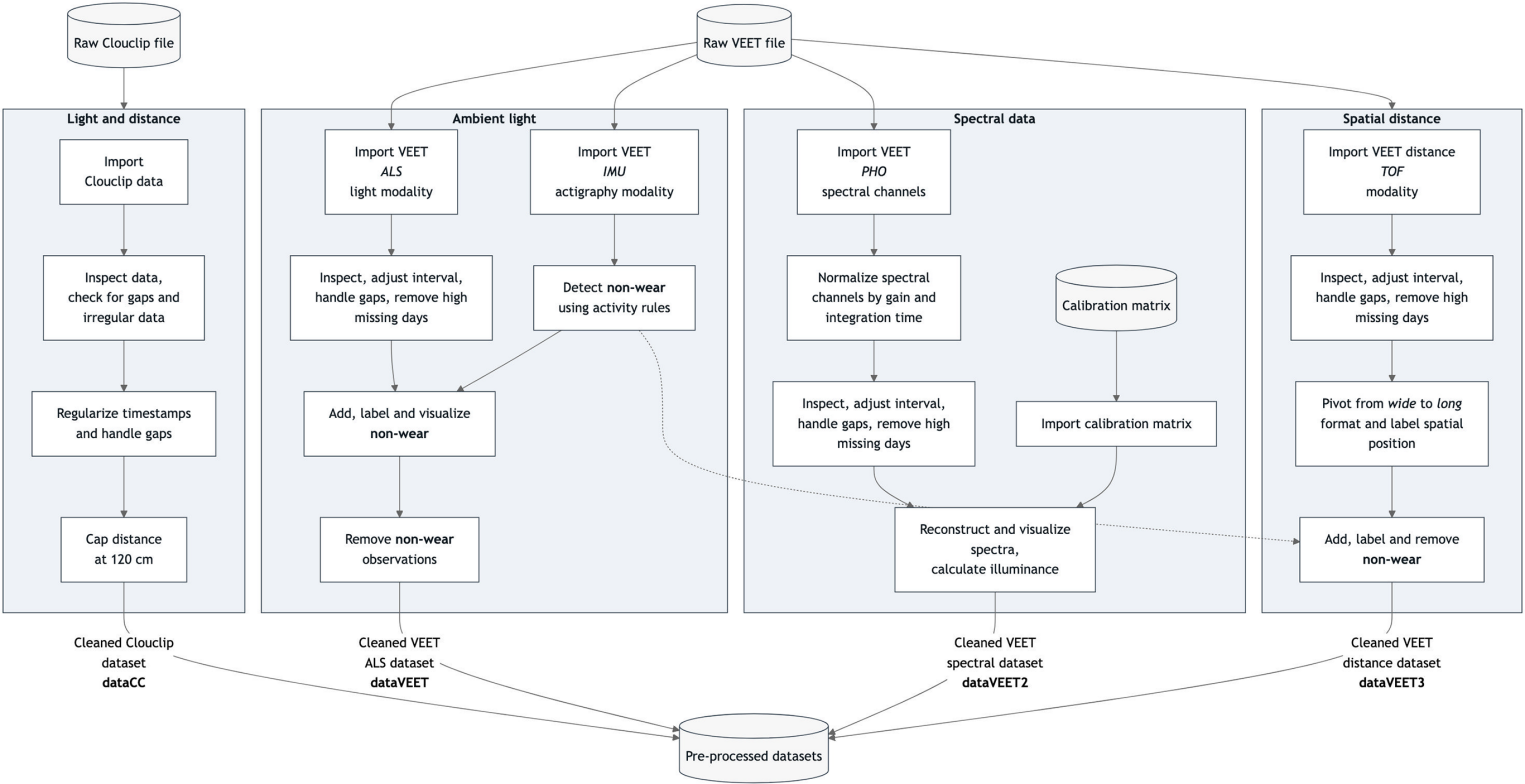
Preprocessing steps in the Supplement 1 document.

The Results section uses imported and preprocessed data from the two devices to calculate metrics. Supplement 1 contains the annotated code and description for the steps involved, which are summarized as follows, and as shown in Figure 1. Please refer to the Supplement 1 for details.

#### Data import

We imported raw data from the Clouclip and VEET devices using LightLogR's built-in import functions, which automatically handle device-specific formats and idiosyncrasies.

The Clouclip export file (provided as a tab-delimited text file) contains timestamped records of distance (centimeters) and illuminance (lux). LightLogR's import$Clouclip function reads this file, after specifying the device's recording timezone, and converts device-specific sentinel codes into proper missing values. For instance, the Clouclip uses special numeric codes to indicate when it is in “sleep mode” or when a reading is out of the sensor's range, rather than recording a normal value. LightLogR identifies -1 (for both distance and lux) as indicating the device's sleep mode and 204 (for distance) as indicating the object was beyond the measurable range, replacing these with NA and logging their status in separate columns. The import routine also provides an initial summary of the dataset, including start and end times and any irregular sampling intervals or gaps. The dataset contained substantial values above the sensible range of a 120-cm distance measurement (Bhandari & Ostrin, 2020), which is why these measurements were set to NA, with an identifier of these cases in the *Dis_status* column.

For the VEET device, data were provided as CSV logs (zipped on GitHub due to size). We focused on the ambient light sensor modality first. Using import$VEET(..., modality = "ALS"), we extracted the illuminance (Lux) data stream and its timestamps. The raw VEET data similarly contain irregular intervals and can contain missing periods (e.g., if the device stopped recording or was reset); the import summary flags these issues.

Besides the Clouclip and VEET, LightLogR 0.10.3 contains import functions for 17 more wearable devices. The package further supports versioning due to evolving data formats and includes documentation for both code-based and codeless additions of new device import functions.

#### Irregular intervals, gaps, and nonwear times

Both datasets showed irregular timing and missing data (i.e., gaps). Irregular data means that some observations did not align with the nominal sampling interval (e.g., slight timing drift or pauses in recording). For the Clouclip 5-second data, we detected irregular timestamps on most days. Handling such irregularities is important because many downstream analyses assume a regular time series. We evaluated strategies to address this, including the following:

• Removing an initial portion of data if irregularities occur mainly during device startup.
• Rounding all timestamps to the nearest regular interval (5 seconds in this case). This is only feasible if no duplicate timestamps are created.
• Aggregating to a coarser time interval (with some loss of temporal resolution). It would also be possible to aggregate to the main interval, in case there are duplicate timestamps from rounding.

Based on the import summary and visual inspection of the time gaps, we chose to round the observation times to the nearest 5-second mark, as this addressed the minor offsets without significant data loss. No duplicate timestamps arose. After rounding timestamps, we added an explicit date column for convenient grouping by day.

We then generated a summary of missing data for each day. Implicit gaps (intervals where the device should have recorded data but did not) were converted into explicit missing entries using LightLogR's gap-handling functions. We also removed days with very little data, focusing on days with substantial wear time. In our Clouclip example, days with less than 1 hour of recordings were dropped. This threshold should be adjusted based on how much complete days matter for a given analysis at hand. For example, in circadian science, the metrics of *interdaily stability* and *intradaily variation* require measurements for each hour of the day.

After these preprocessing steps, the Clouclip dataset had no irregular timestamps remaining and contained explicit markers for all periods of missing data (e.g., times when the device was off or not worn). The distance and illuminance values were now ready for metric calculations. Because the device was put in sleep mode when not worn, there are no measurements during nonwear times. As we are mostly using the Clouclip data for its distance recordings, even short periods are useful.

The VEET illuminance data underwent a similar cleaning procedure. To make the VEET's 2-second illuminance data more comparable to the Clouclip's and to reduce computational load, we aggregated the illuminance time series to 5-second intervals. Aggregation was performed with the arithmetic mean of values in a 5-second bin. We then inserted explicit missing entries for each whole day and removed days with more than 1 hour of missing illuminance data. After cleaning, 6 days of VEET illuminance data with good coverage remained for analysis (see Supplement 1 for details).

Finally, for spectral analysis, we imported the VEET's spectral sensor modality and, for the distance analysis, the time-of-flight modality. This required additional processing: The raw spectral data consist of counts from nine wavelength-specific channels (approximately 415 nm through 910 nm, unequally spaced between 30 and 50 nm, plus one broadband clear channel covering the whole range of individual channels, another broadband channel for flicker detection, and a dark channel), along with a sensor gain setting. We aggregated the spectral data to 5-minute intervals to focus on broader trends and reduce data volume. Each channel's counts were normalized by the appropriate gain. Using a calibration matrix provided by the manufacturer (specific to the spectral sensor model), we reconstructed full spectral power distributions for each 5-minute interval. The end result is a list column in the dataset where each entry is the estimated spectral irradiance across wavelengths for that observation. Detailed spectral preprocessing steps, including the calibration and normalization, are provided in Supplement 1. After spectral reconstruction, the dataset was ready for calculating spectrum-based metrics.

Similarly, the time-of-flight modality contains 256 values per observation, encoding an 8 × 8 grid of distance and confidence measurements for up to two objects (8 × 8 grid, times two objects, times distance + confidence column for each object and grid point → 256 values). For computational reasons, only the first object was kept. These data were pivoted into a long format, where each row contains the distance and confidence data for a given position in the grid and a given datetime. After pivoting and converting grid positions into a deviation angle from central view, the dataset was ready to be used for distance analysis.

Because the VEET devices record even when not worn, a nonwear detection using the devices’ actigraphy modality was implemented. This process used the standard deviation of a linear motion sensor in a 5-minute bin with a visually derived threshold to separate wear from nonwear time. Measurements of illuminance and distance were consequently removed during the calculated nonwear times.

This tutorial will start by importing a Clouclip dataset and providing an overview of the data. The Clouclip export is considerably simpler compared to the VEET export, containing only Distance and Illuminance measurements. The VEET dataset will be imported later for the spectrum-related metrics.


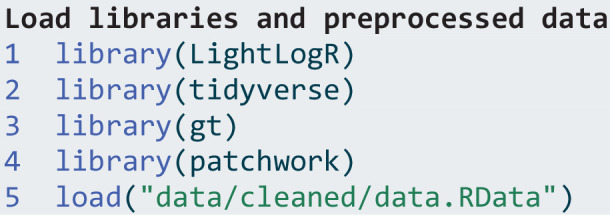



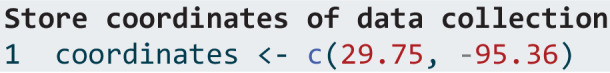

**Line 1** Coordinates for Houston, Texas; coordinates are important to calculate and visualize photoperiods later

## Results

**Figure 2.**
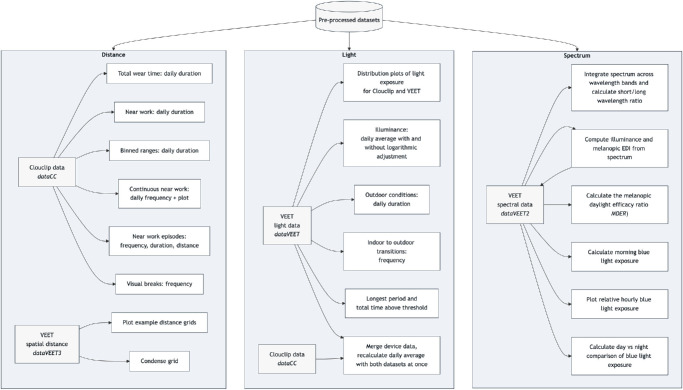
Workflows that are covered in the Results section. The preprocessed datasets are covered in detail in the Supplement 1.

Figure 2 shows an overview of the covered workflows in the Results section.

We first examine metrics related to viewing distance, using the processed Clouclip dataset. Many distance-based metrics are computed for each day and then averaged over weekdays, weekends, or across all days. To facilitate this, we define a helper function that will take daily metric values and calculate the mean values for weekdays, weekends, and the overall daily average.

For longer code cells, core functionality may be highlighted by graying out less relevant lines.


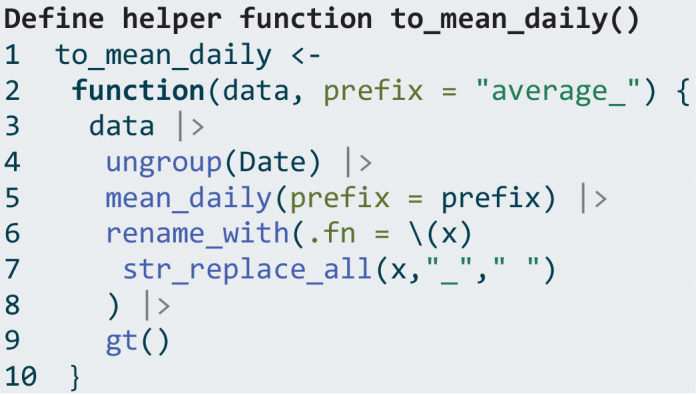

**Line 4** Ungroup “by days”
**Line 5** Calculate the averages per grouping
**Line 6–8** Remove underscores in names
**Line 9** Format as a gt table for display

### Total wear time daily

*Total wear time daily* refers to the amount of time the device was actively collecting distance data each day (i.e., the time the device was worn and operational). We compute this by summing all intervals where a valid distance measurement is present, ignoring periods where data are missing or the device was off. The results are shown in Table 3.

**Table 3.** Total wear time per day (average across days).

	Total wear duration
Mean daily	55,430 s (∼15.4 hours)
Weekday	54,221 s (∼15.06 hours)
Weekend	58,452 s (∼16.24 hours)


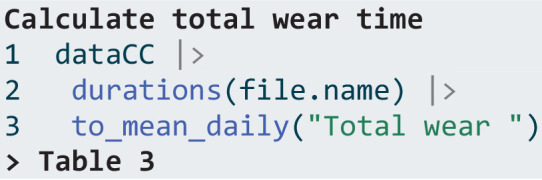

**Line 2** Calculate total duration of data per day, setting file.name as the column in question only gives us data points that were included in the original file
**Line 3** Using the helper function defined above

We see there is about an hour more data per day on the weekend compared to during weekdays.

### Duration within distance ranges

Many myopia-relevant metrics concern the time spent at certain viewing distances (e.g., “near work” vs. intermediate or far distances). We calculate the duration of time spent in specific distance ranges. Table 4 shows the average daily *duration of near work*, defined here as time viewing at 15 to 60 cm. Table 5 provides a more detailed breakdown across multiple distance bands.

**Table 4.** Daily duration of near work (15- to 60-cm viewing distance).

	Near-work duration
Mean daily	12,274 s (∼3.41 hours)
Weekday	11,803 s (∼3.28 hours)
Weekend	13,450 s (∼3.74 hours)

**Table 5.** Daily duration in each viewing distance range in minutes. For better readability, the table was transposed (compared to the code results).

	Mean daily, min	Weekday, min	Weekend, min
Far	136	157	83
Far intermediate	17	17	16
Moderately intermediate	30	31	26
Intermediate	22	23	22
Near intermediate	24	23	27
Moderately near	22	19	30
Near	35	33	40
Fairly near	40	40	38
Very near	67	64	74
Extremely near	32	30	36

#### Duration of near work


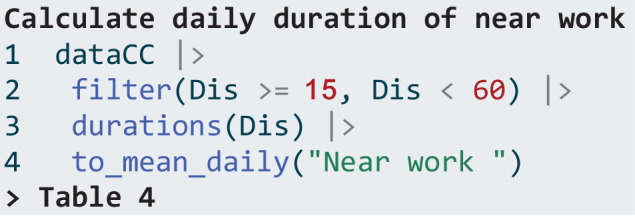

**Line 2** Consider only distances in [15, 60) cm
**Line 3** Total duration in that range per day

We see that, on average, there are about 3.4 hours of near work per day, with about 30 minutes more near time on weekends than on weekdays. As mentioned above under Methods, these data were collected in a group of students during the COVID-19 pandemic (November 2020). This means that the underlying behavioral patterns, day to day, might not reflect typical differences in behavior between weekdays and weekends. This holds true for the results in the following sections as well.

#### Duration within distance ranges

First, we define a set of distance breakpoints and descriptive labels for each range. Depending on the type of device (valid measurement range) and theoretical assumptions, these could be chosen differently by the user:


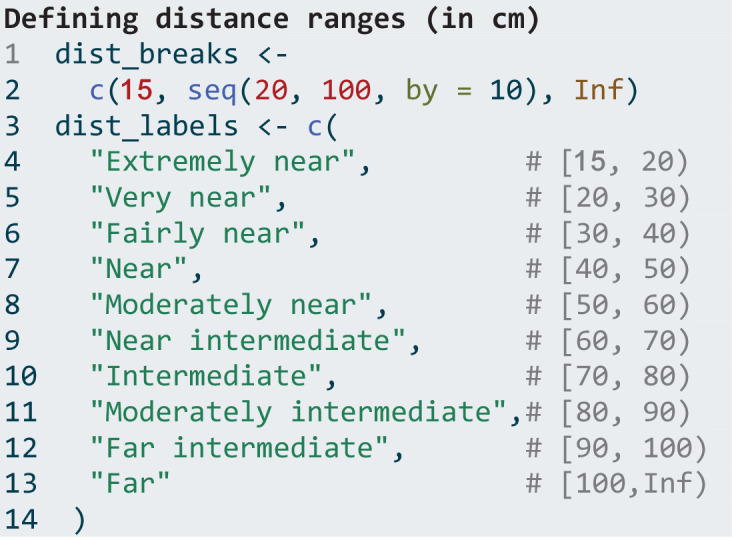


Now we cut the distance data into these ranges and compute the daily duration spent in each range. As we set values above 120 cm to NA in the preprocessing stage (see Supplement 1), these would be missing from this analysis. However, we know that a value of, for example, 180 cm, while not accurate to within a few centimeters, is almost certainly greater than 100 cm. Thus, we will create a column that sets these values to 120 cm. As long as only duration and not average distance is required, this remains valid.


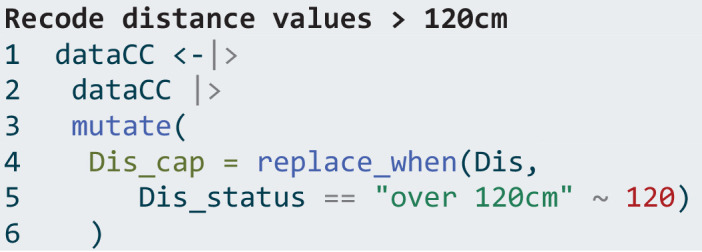



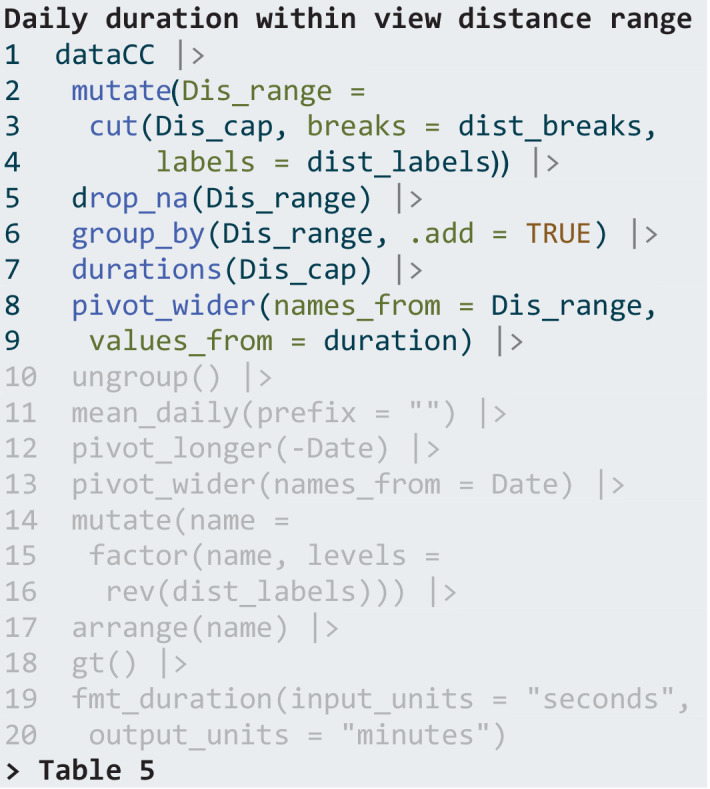

**Lines** **2–4** Categorize distances
**Line 5** Remove observations with no data
**Line 6** Group by distance range (in addition to the date)
**Line 7** Duration per range per day
**Lines 8–9** Pivot data from long to wide format (ranges as columns)

**Figure 3.**
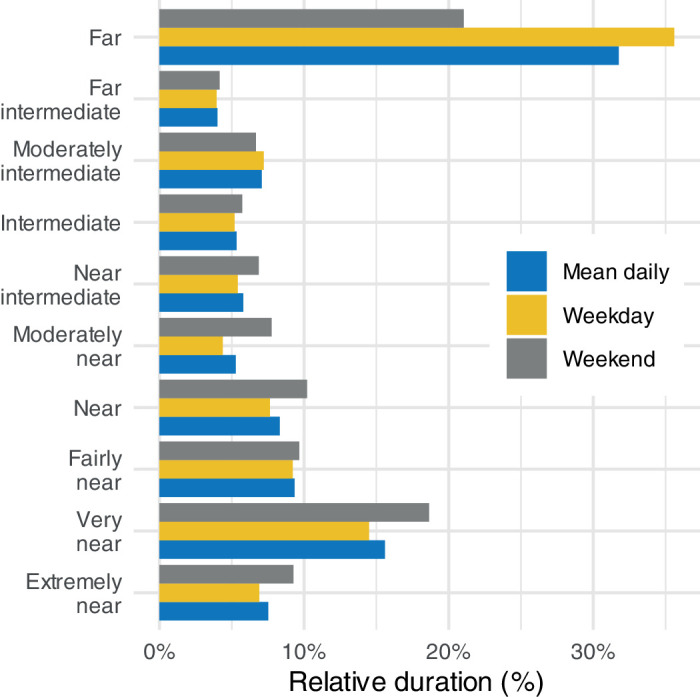
Percentage of total time spent in each viewing distance range for an average day (mean daily), average weekday, or weekend.

To visualize this, Figure 3 illustrates the relative proportion of time spent in each distance range.

### Frequency of continuous near work

Continuous near work can be understood as sustained viewing within a near distance for some minimum duration, allowing only brief interruptions. We use LightLogR's cluster function to identify episodes of continuous near work. Here, we define a near-work episode as a viewing distance between 20 and 60 cm that lasts at least 15 minutes, with interruptions of up to 30 seconds allowed (meaning short breaks ≤30 seconds do not end the episode). Using extract_clusters() with those parameters, we count how many such episodes occur per day.

**Figure 4.**
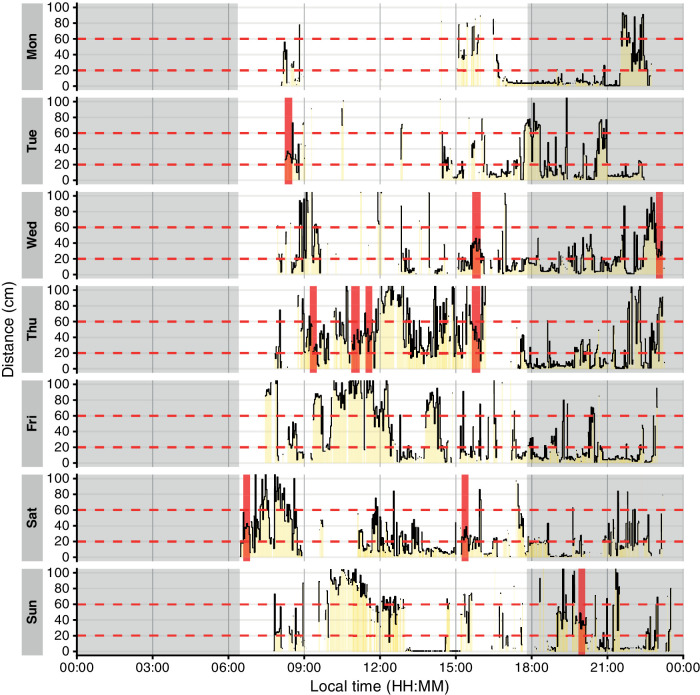
Example of continuous near-work episodes. Red shaded areas indicate periods of continuous near work (20–60 cm for ≥15 minutes, allowing ≤30-second interruptions). Black trace is viewing distance over time of day; red dashed lines mark the 20-cm and 60-cm boundaries. Gray shaded areas show nighttime between civil dusk and civil dawn.


Table 6 summarizes the average frequency of continuous near-work episodes per day, and Figure 4 provides an example visualization of these episodes on the distance time series.

**Table 6.** Frequency of continuous near-work episodes per day.

	Frequency of episodes
Mean daily	1.43
Weekday	1.40
Weekend	1.50

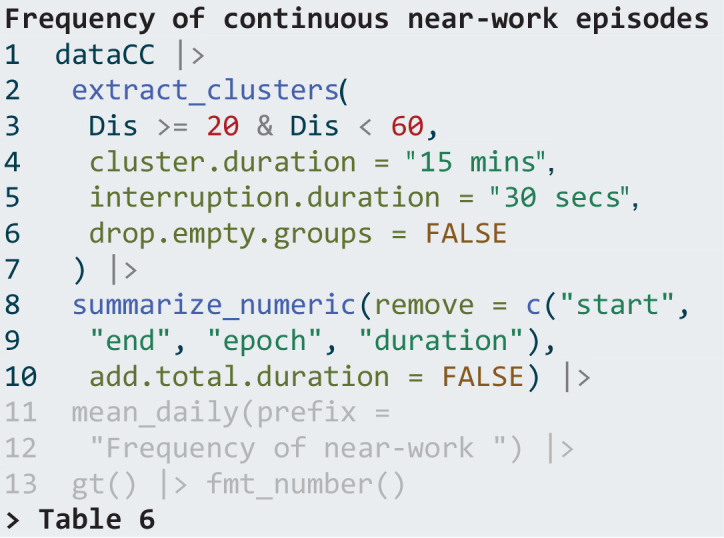

**Line 3** Condition: near-work distance
**Line 4** Minimum duration of a continuous episode
**Line 5** Maximum gap allowed within an episode
**Line 6** Keep days with zero episodes in output
**Lines 8–10** Count number of episodes per day

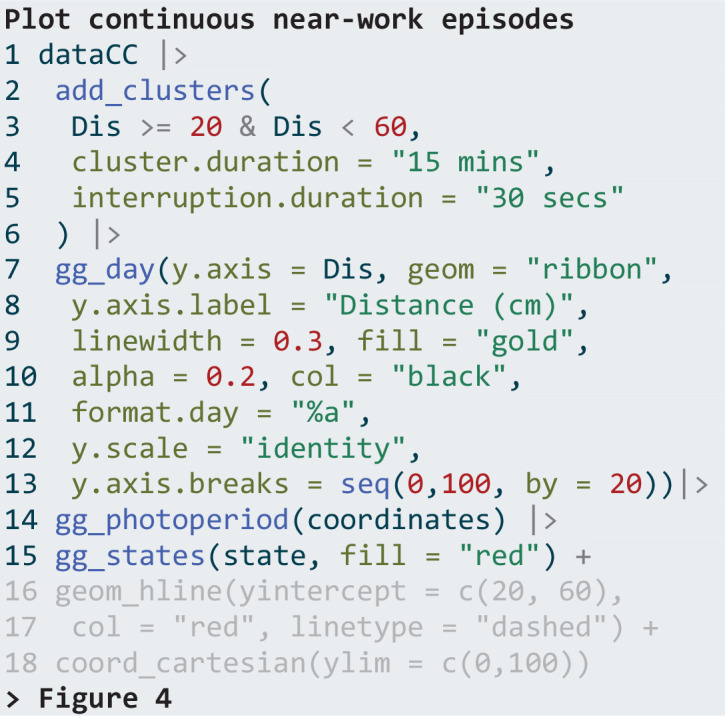

**Lines 3** **–** **5** As in code cell above
**Line 11** Label weekdays (*%a)* instead of dates (default: *%d/%m*)
**Line 14** Add photoperiod information
**Line 15** Add state bands

### Near-work episodes

Beyond frequency, we can characterize near-work episodes by their duration and typical viewing distance. This section extracts all near-work episodes (using a 5-second minimum duration to capture more routine near-work bouts) and summarizes three aspects:

1. frequency (count of episodes per day),
2. average duration of episodes, and
3. average distance during those episodes.

These results are combined in Table 7.

**Table 7.** Near-work episodes: mean duration, and mean viewing distance number of episodes.

	Duration	Distance, cm	Episodes
Mean daily	210 s (∼3.5 minutes)	36	55
Weekday	231 s (∼3.85 minutes)	35	44
Weekend	157 s (∼2.62 minutes)	39	81


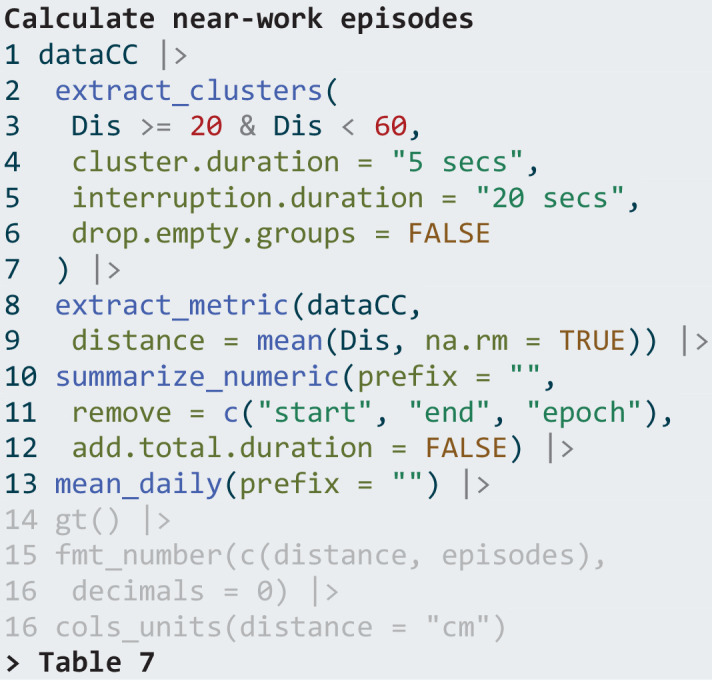

**Line 4** Minimal duration to count as an episode (set to interval level of dataCC)
**Lines 8–9** Calculate mean distance during each episode
**Lines 10** **–** **12** Calculate averages for all numeric columns per group
**Line 12** Daily averages for each metric

In the code cell above, extract_metric(..., distance = mean(Dis, ...)) computes the mean viewing distance during each episode, and the subsequent summarize_numeric and mean_daily steps derive daily averages of episode count, duration, and distance.

### Visual breaks

**Figure 5.**
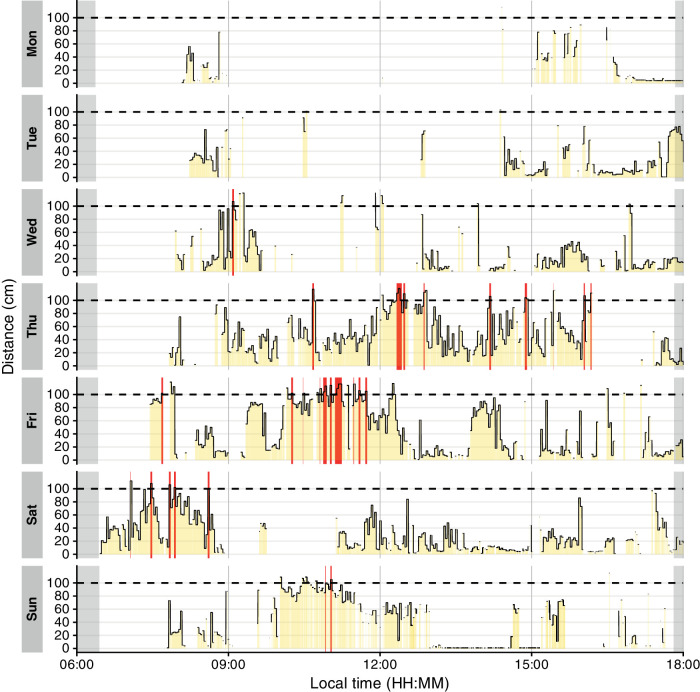
Plot of visual breaks (red vertical bars). Black traces show distance measurement data. Gray shaded areas show nighttime between civil dusk and civil dawn (photoperiod).


Visual breaks, as defined in this article, require a minimum break length, and the previous episode is important. This leads to a two-step process, where we first extract instances of Distance above 100 cm for at least 20 seconds, before we filter for a previous (valid) duration of at maximum 20 minutes. Table 8 provides the daily frequency of visual breaks, and Figure 5 shows when these occur. While visual breaks are not a commonly used metric, they highlight the powerful detection mechanisms that are available.


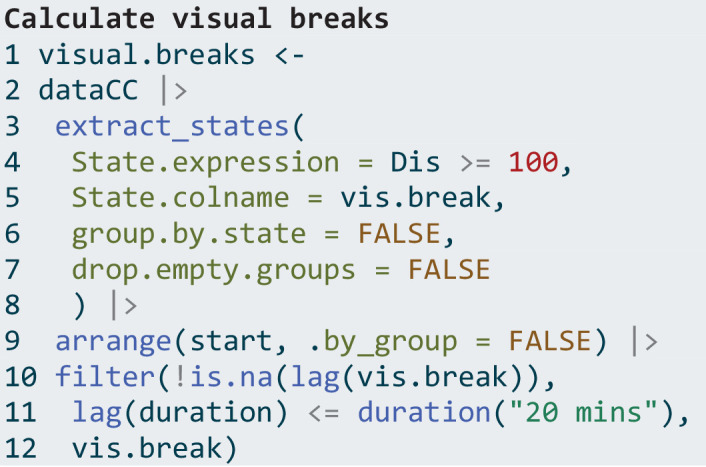

**Line 3–8** Define the condition, greater 100 cm away, and store it in a variable vis.break. Empty groups (days), should not be dropped, and the new state should not be a new grouping variable.
**Line 9** Because *extract_states()* sorts the output by the new states, we need to arrange them in timely order
**Line 10** Only keep instances where the previous condition is not *NA* (i.e., we do not know whether it is below or above 100 cm distance)
**Line 11** Only keep instances, where the previous conditions is at max 20 minutes
**Line 12** Only keep instances, where the current condition is equal to or greater 100 cm distance

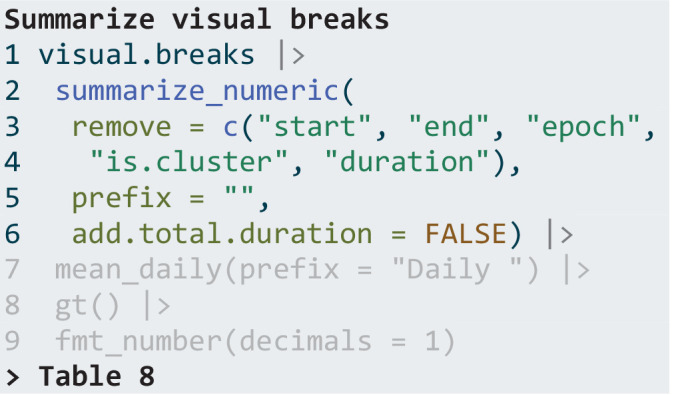

**Line 1** Use the previously calculated visual.breaks
**Lines 2–5** Count the number of episodes

**Table 8.** Frequency of visual breaks (*n* per day).

	Daily episodes
Mean daily	4.1
Weekday	4.2
Weekend	4.0

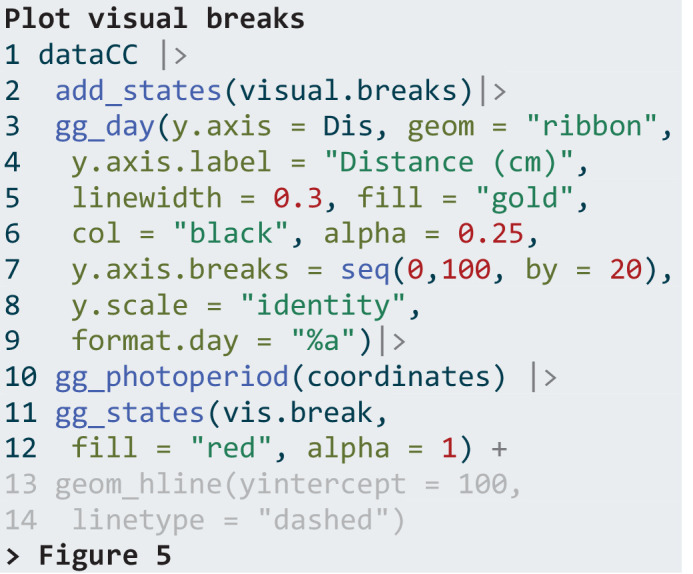

**Line 2** Add the previously calculated visual.breaks to the data
**Line 10** Add photoperiod information to the plot
**Lines 11–12** Highlight the visual breaks

### Distance with spatial distribution

**Figure 6.**
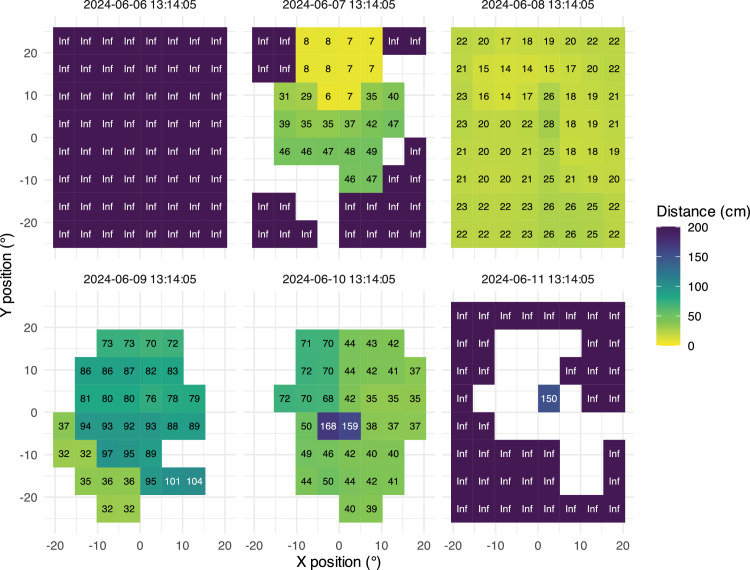
Example observations of the measurement grid at 1:14 p.m. for each measurement day. Text values show distance in centimeters. Empty grid points show values with low confidence. Zero-distance values were replaced with infinite distance and plotted despite low confidence.

The Clouclip device outputs a singular measure for distance, while the visual environment in natural conditions contains many distances, depending on the solid angle and direction of the measurement. A device like the VEET increases the spatial resolution of these measurements, allowing for more in-depth analyses of the size and position of an object within the field of view. In the case of the VEET, data are collected from an 8 × 8 measurement grid, spanning 52° vertically and 41° horizontally. Figure 6 shows sample observations from six different days at the same time.


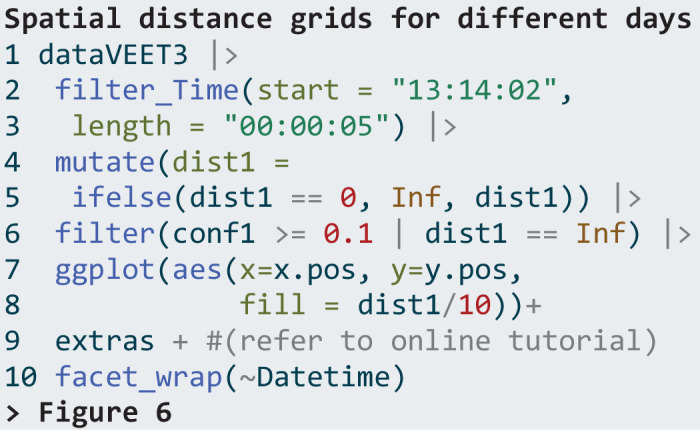

**Lines 2–3** Choose a particular observation
**Lines 4–5** Replace 0 distances with infinity
**Line 6** Remove data that has less than 10% confidence
**Lines 7–9** Plot the data. For the full code (including the contents of extras, please refer to the online tutorial)
**Lines 10** Show one plot per day

This spatial resolution necessitates a change in the data format. When we try to apply the preprocessed VEET data to the functions described above for the Clouclip device, we run into issues. This is because each time point is repeated 64 times to include as many spatial positions. LightLogR expects unique time points for all observations within each group (in our case, days within participants). To use these distance data in the framework shown above for the Clouclip device, a sensible method to condense the data has to be applied. There are many ways in which a spatially resolved distance measure could be utilized for analysis:

• Where in the field of view are objects in close range?
• How large are near objects in the field of view?
• How varied are distances within the field of view?
• How close are objects/is viewing distance in a region of interest within the field of view?

Possible methods include the following:

• Average across all (high confidence) distance values within the grid
• Closest (high confidence) distance within the grid
• (High confidence) values at or around a given grid position, for example, ±10 degrees around the central view (0°)

Many more options are available based on the spatial dataset (e.g., condensation rules based on the number of points in the grid with a given condition or the variation within the grid).

We will demonstrate these three methods for a single day (2024-06-10), all leading to a data structure akin to the Clouclip (i.e., to be used for further calculation of visual experience metrics).


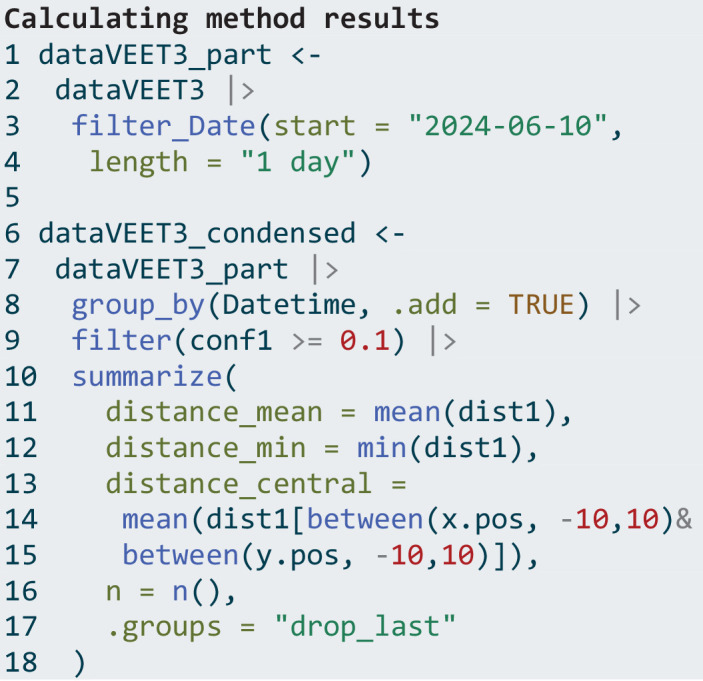

**Lines 3–4** Filter one day
**Line 8** Group additionally by every observation (Datetime)
**Line 9** Remove data with low confidence
**Line 11** Average across all distance values
**Line 12** Closest across all distance values
**Line 13–15** Central distance
**Line 16** Number of (valid) grid points

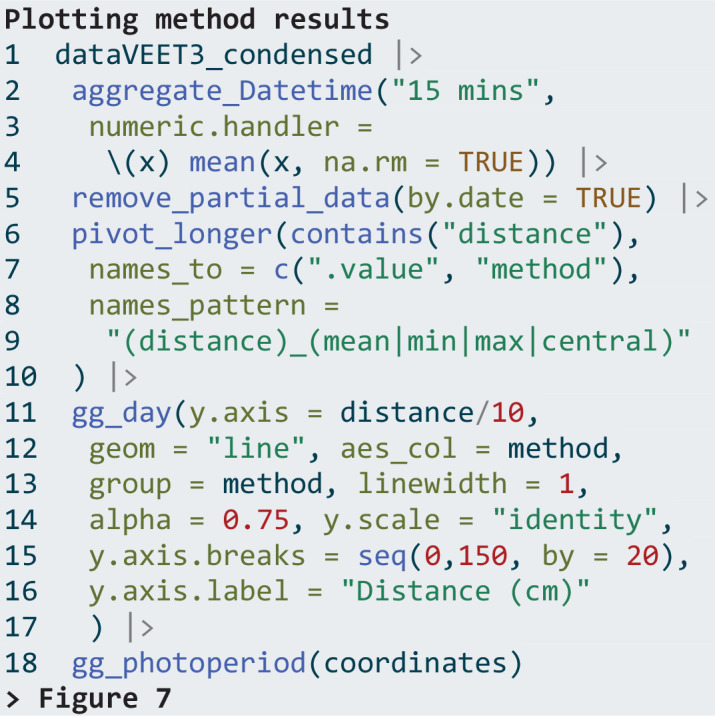

**Lines 2–4** Aggregate to 15 minute data
**Line 5** Remove data points that fall exactly on midnight of the following day
**Lines 6–10** Pivoting the method results from wide to long for plotting
**Lines 11–18** Plotting function

**Figure 7.**
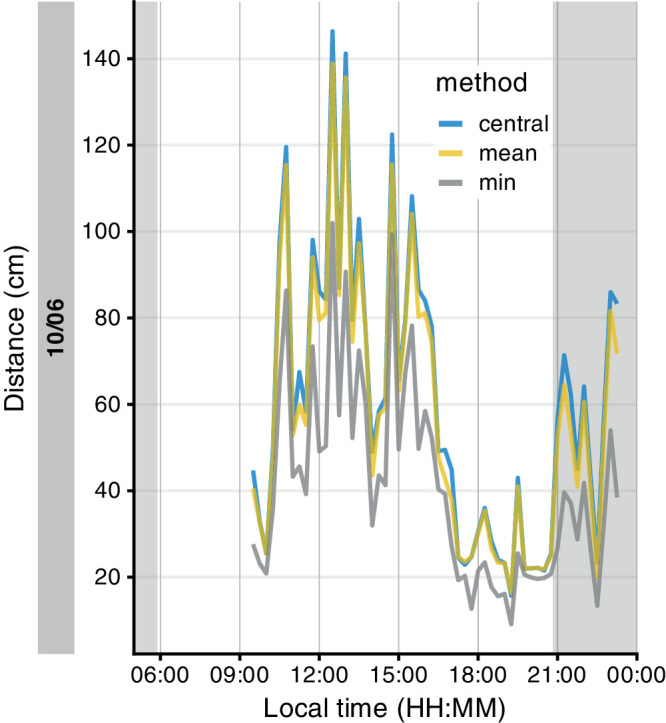
Comparison of condensation methods for a spatial grid of distance measurements. The lines represent an average across all data points (yellow), the minimum distance (gray), or the central 10° (blue). Data points with confidence less than 10% were removed prior to calculation.

As can be seen in Figure 7, while the overall pattern is similar regardless of the used method, there are notable differences between the methods, which will consequently affect downstream analyses. Most importantly, the process of condensation has to be well documented and reproducible, as shown above. Any of these data could now be used to calculate the frequency of continuous near work, visual breaks, or near-work episodes as described above.

### Light

To illustrate light exposure metrics, we turn to a different dataset, this one taken from the VEET device's illuminance data, which capture a broader range of lighting conditions (although both device types are able to capture broadly the same range of illuminance). We import the VEET ambient light data (already preprocessed to have regular 5-second intervals as described above) and briefly examine its distribution.

**Figure 8.**
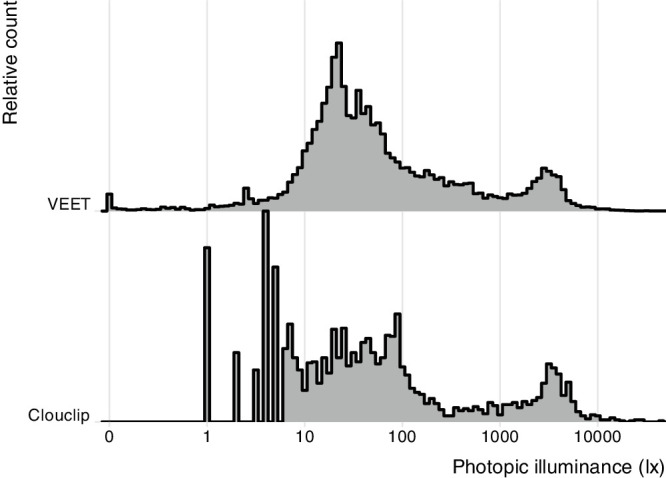
Histogram of illuminance values from the VEET and Clouclip dataset (5-second data). Indoor light exposure mainly occurs around 10^1^ to 10^2^ lx. Outdoor (day)light exposures in bright conditions are mostly around 10^3^ to 10^4^ lx.

**Illuminance distribution:** The VEET (Figure 8) data include indoor and outdoor exposures up to several thousand lux. The contrast between the two settings is evident from comparing histograms of the two datasets’ lux values (Clouclip and VEET), where the main peak is similarly positioned between 10 and 100 lx, but the tails differ. The VEET illuminance histogram (see Figure 8) shows a heavily skewed distribution with a considerable number of zero lx values (indicating intervals of complete darkness or the sensor being covered) and a long tail extending to very high lux values. Such zero-inflated and skewed data are common in wearable light measurements (Zauner, Guidolin, & Spitschan, 2025). The Clouclip, however, shows a minimal value of 1 lx and worse discrimination below 10 lx (see Figure 8). Additionally, the Clouclip was not recording when the device was not worn, for example, during sleep (sleep mode), compared to the continuous recording of VEET devices, where nonwear data were removed in the preprocessing (see Supplement 1), based on the activity sensors of the device.

After confirming that the VEET data cover a broad dynamic range of lighting, we proceed with calculating light exposure metrics. (The VEET data had been cleaned for gaps and irregularities as described earlier, and nonwear times were removed; see Supplement 1 for the details.)

#### Average light exposure

A basic metric is the average illuminance over the day. Table 9 shows the mean illuminance (in lux) for weekdays, weekends, and the overall daily mean, calculated directly from the raw lux values.

**Table 9.** Mean light exposure (illuminance) per day.

	Mean photopic illuminance (lx)
Mean daily	481.8
Weekday	538.1
Weekend	341.1


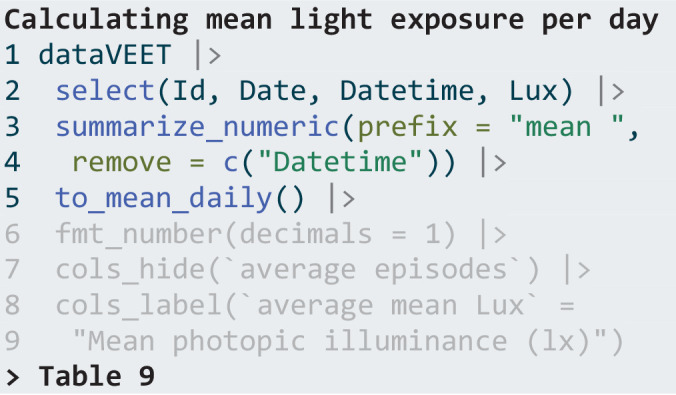


However, because illuminance data tend to be extremely skewed and contain many zero values (periods of darkness), the arithmetic mean can be misleading. A common approach is to apply a logarithmic transform to illuminance before averaging, which down-weights extreme values and accounts for the multiplicative nature of light intensity effects. LightLogR provides helper functions log_zero_inflated() and its inverse exp_zero_inflated() to handle log-transformation when zeros are present (by adding a small offset before log and back-transforming after averaging). Using this approach, we recompute the daily mean illuminance. The results in Table 10 show that the log-transformed mean (back-transformed to lux) is much lower, reflecting the fact that for much of the time, illuminance was near zero. This transformed mean is often more representative of typical exposure for skewed data.

**Table 10.** Mean light exposure per day (after logarithmic transformation to account for zero inflation and skewness).

	Mean photopic illuminance (lx)
Mean daily	57.0
Weekday	70.1
Weekend	33.9


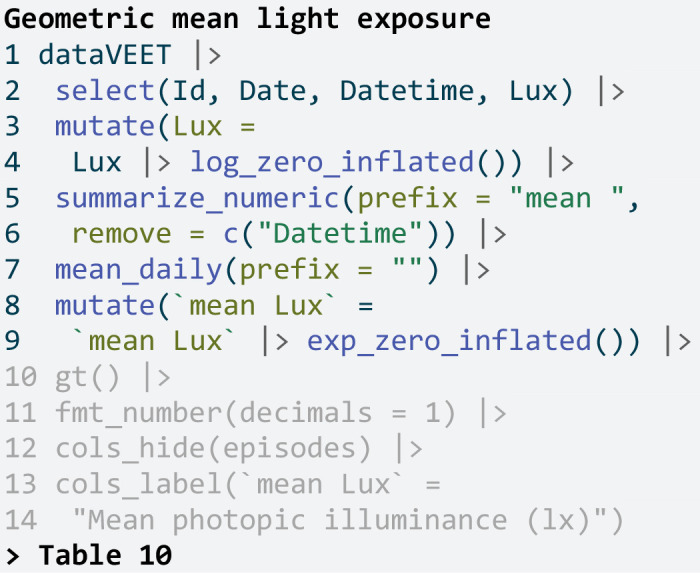

**Lines 3–4** Log transform with zero handling (base 10)
**Lines 5–7** Calculate daily mean of log-lux
**Lines 8–9** Back-transform to lux

#### Duration in high-light (outdoor) conditions

Another important metric is the amount of time spent under bright light, often used as a proxy for outdoor exposure. We define thresholds corresponding to outdoor light levels (e.g., 1,000 lx and above). Here, we categorize each 5-second interval of illuminance into bands: outdoor bright (≥1,000 lx), outdoor very bright (≥2,000 lx), and outdoor extremely bright (≥3,000 lx). We then sum the duration in each category per day.

**Figure 9.**
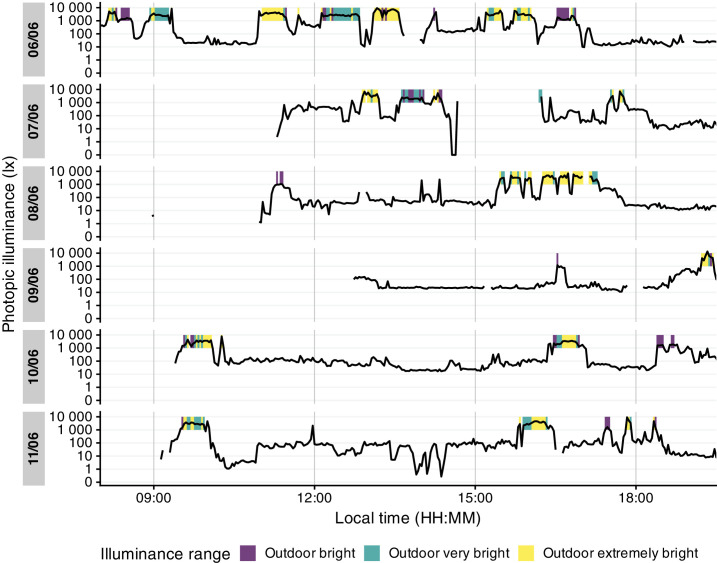
Outdoor light exposure over time with a 2-minute interval. Colored bands indicate periods when illuminance exceeded outdoor thresholds for at least half of each interval: violet for ≥1,000 lx, green for ≥2,000 lx, and yellow for ≥3,000 lx.


While daylight levels can far exceed the recorded light levels, those are usually recorded with direct sunlight and without obstruction. Under normal viewing conditions, at eye level, and avoiding glare, daylight levels of a few thousand lux are at the higher end of the distribution (Murukesu, Zauner, & Spitschan, 2025). Figure 9 shows a bimodal distribution, with the right mode representing outdoor lighting conditions. In a 2023 review of light dosimeters to investigate the light–myopia relationship (Hönekopp & Weigelt, 2023), 1,000 lx was the predominant cutoff value to distinguish indoor versus outdoor environments. It is not, however, without critique, and other thresholds (Patterson Gentile et al., 2025) and classification methods are proposed (Tabandeh & Spitschan, 2025).

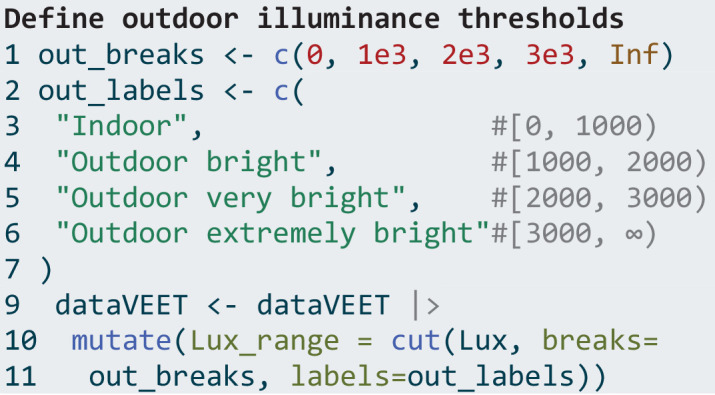


Now we compute the mean daily duration spent in each of these outdoor light ranges (Table 11):

**Table 11.** Average daily duration in outdoor-equivalent light conditions (column order is different from code output).

Outdoor	Bright, min	Very bright, min	Extremely bright, min	Indoor, min
Mean daily	24	28	42	663
Weekday	29	35	48	688
Weekend	10	10	28	602


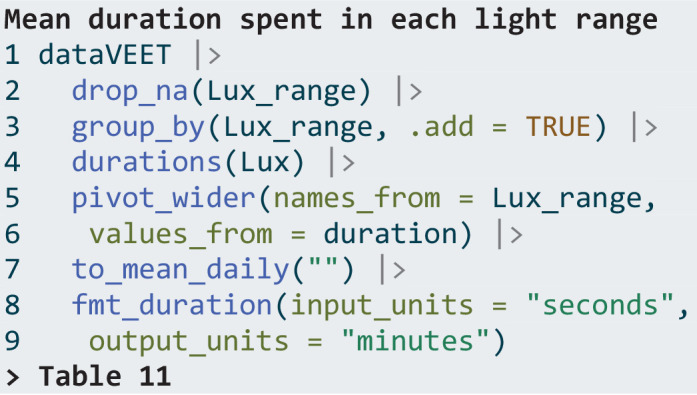


It is also informative to visualize when these high-light conditions occurred. Figure 9 shows a timeline plot with periods of outdoor-level illuminancehighlighted in color. In this example, violet denotes ≥1,000 lx, green ≥2,000 lx, and yellow ≥3,000 lx. (to dawn) for context.


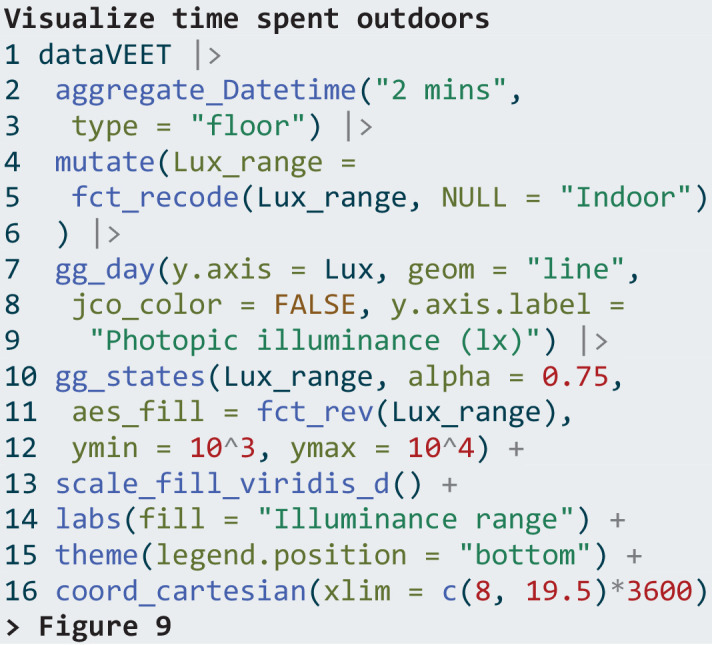

**Lines 2–3** Aggregating data to 5-minute bins
**Lines 4–6** Removing the indoor condition
**Lines 7–9** Setting up the basic plot
**Lines 10–2** Adding state information on the illuminance ranges
**Line 16** Setting the x-axis limits to cover daytime hours

#### Frequency of transitions from indoor to outdoor light

We next consider how often the subject moved from an indoor light environment to an outdoor-equivalent environment. We operationally define an “outdoor transition” as a change from <1,000 lx to ≥1,000 lx. Using the cleaned VEET data, we extract all instances where illuminance crosses that threshold from below to above.

Table 12 shows the average number of such transitions per day. Note that if data are recorded at a fine temporal resolution (5 seconds here), very brief excursions above 1,000 lx could count as transitions and inflate this number. Indeed, the initial count is fairly high, reflecting fleeting spikes above 1,000 lx that might not represent meaningful outdoor exposures.

**Table 12.** Average daily count of transitions from indoor (<1,000 lx) to outdoor (≥1,000 lx) lighting when looking at 5-second epochs.

	Mean epoch, s	Episodes
Mean daily	5	64
Weekday	5	72
Weekend	5	46


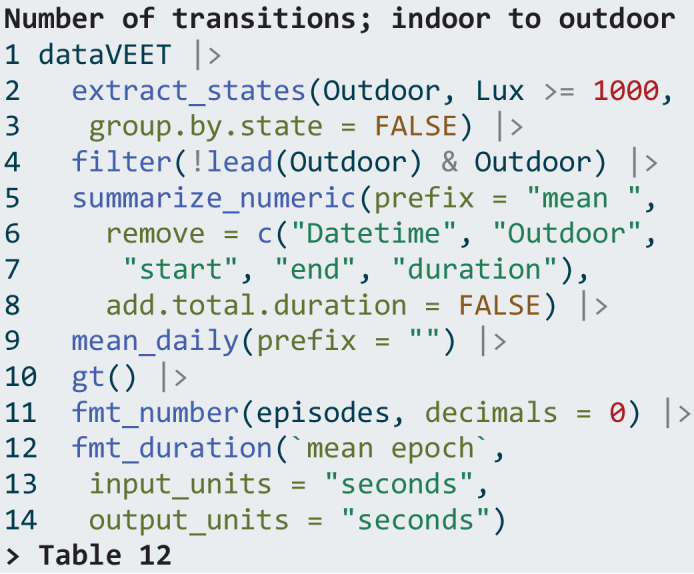

**Lines 2–3** Label each interval as Outdoor (Lux ≥ 1000) or not
**Line 4** Find instances where the previous interval was “indoor” and current is “outdoor”

To obtain a more meaningful measure, we can require that the outdoor state persists for some minimum duration to count as a true transition (filtering out momentary fluctuations around the 1,000-lx mark). For example, we can require that once ≥1,000 lx is reached, it continues for at least 5 minutes (allowing short interruptions up to 20 seconds). Table 13 applies this criterion, resulting in a lower, more plausible transition count.

**Table 13.** Daily indoor-to-outdoor transition count (requiring ≥5-minute duration of ≥1,000 lx to count).

	Mean epoch, s	Episodes
Mean daily	5	5
Weekday	5	6
Weekend	5	4


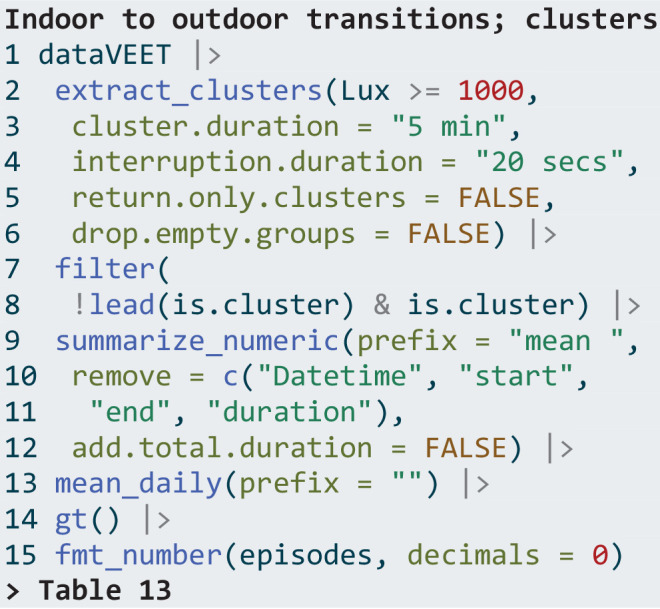


**Figure 10.**
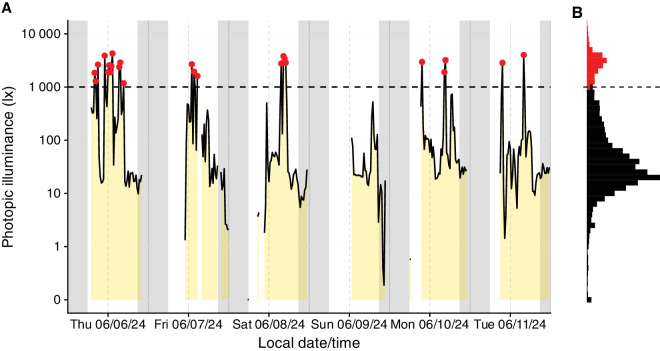
(**A**) Outdoor light exposure over time with a 20-minute interval. Gray shaded regions denote night (from civil dusk to dawn). Red points denote intervals with a median illuminance above 1,000 lx. (**B**) Histogram of 5-second data (colors as in panel A).


Another possibility would be to check for stronger changes between consecutive episodes or aggregate the data further for average values across longer time spans (see Figure 10).

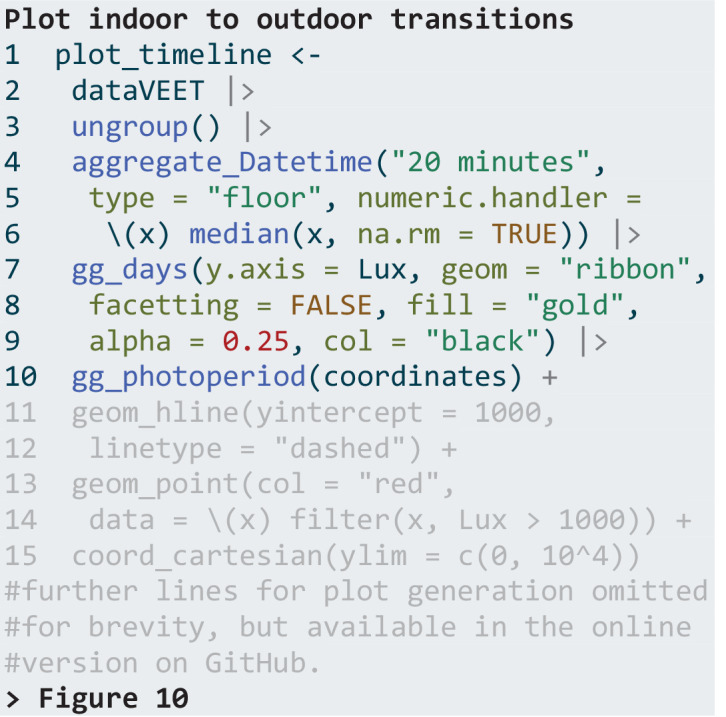

**Lines 4–6** Function to reduce the interval through aggregation (here 20 minute bins). type = “floor” specifies that values are always sorted in the next lower bin. This avoids rollovers around midnight into the next day. The function provides sensible default handlers for data types like numeric, text, factor, boolean, etc. For numeric, the default is mean, and NAs propagate. Here, we set the numeric.handler to the median and ignore missing values.

#### Longest sustained bright-light period

The final light exposure metric we illustrate is the longest continuous period above a certain illuminance threshold (often termed longest period above threshold, e.g., PAT1000 for 1,000 lx). This gives us a sense of the longest outdoor exposure in a day. Along with it, one might report the total duration above that threshold in the day (TAT1000). While we could derive these from the earlier analyses, LightLogR provides dedicated metric functions for such calculations, which can compute multiple related metrics at once.

Using the function period_above_threshold() for PAT and duration_above_threshold() for TAT, we calculate both metrics for the 1,000-lx threshold. Table 14^15^,^16^ shows the mean of these metrics across days (i.e., average longest bright period and average total bright time per day).

**Table 14.** Longest period and total duration above 1,000 lx (PAT1000 and TAT1000).

	Period above 1,000 lx	Duration above 1,000 lx
Mean daily	1,208 s (∼20.13 minutes)	5,703 s (∼1.58 hours)
Weekday	1,469 s (∼24.48 minutes)	6,815 s (∼1.89 hours)
Weekend	555 s (∼9.25 minutes)	2,922s (∼48.7 minutes)

**Table 15.** Overview of the merged dataset.

	Mean	Min	Max
Participants (*n*)	2		
Participant-days (*n*)	13	6	7
Days ≥50% complete (*n*)	10	3	7
Missing/irregular (%)	42	32	59

**Table 16.** Recalculation of the mean light exposure per day (after logarithmic transformation to account for zero inflation and skewness) with the merged dataset.

	Mean photopic illuminance (lx)
Clouclip	
Mean daily	45.5
Weekday	55.8
Weekend	27.3
VEET	
Mean daily	57.0
Weekday	70.1
Weekend	33.9


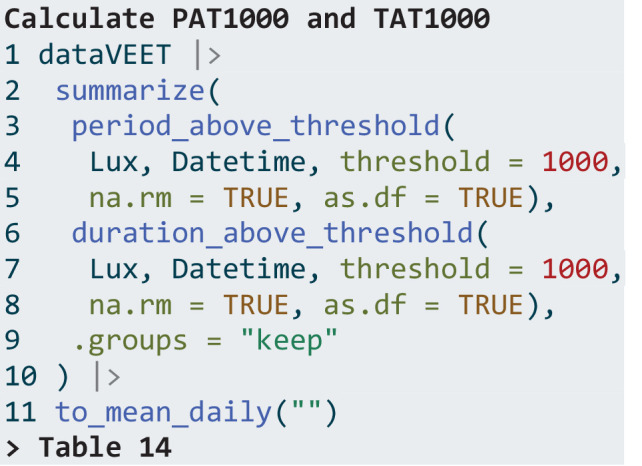


#### Merging data streams

Note that while imports from different devices can be merged, devices differ in their sensors, electronics, housing or diffuser form factors, and on-device data-processing pipelines. All of these factors affect the comparability of measurements, even when devices output the same variable (e.g., illuminance or distance). If data from different devices with the same measurement variable are to be merged, the corresponding variable names should be standardized beforehand—for example, renaming Lux and LIGHT to illuminance. If we wanted to analyze the VEET data together with the Clouclip data, for example, we would not have to rename anything, as both carry their illuminance measurements in the variable Lux. The following example shows how the combination of datasets would lead to a combined dataset (Table 15) and how that would affect analysis outcomes. It is the responsibility of the researcher to perform device calibration and/or checks for a similar measurement fidelity.


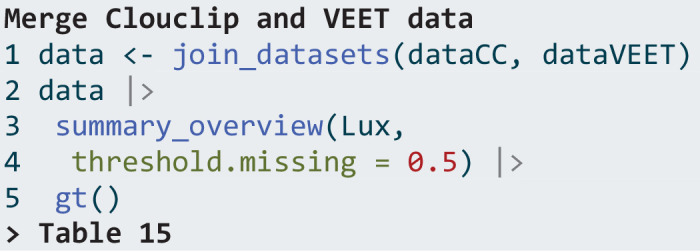


We will reuse the example from *Average light exposure*, but instead of one participant, we now have data from two devices and participants (Table 16).


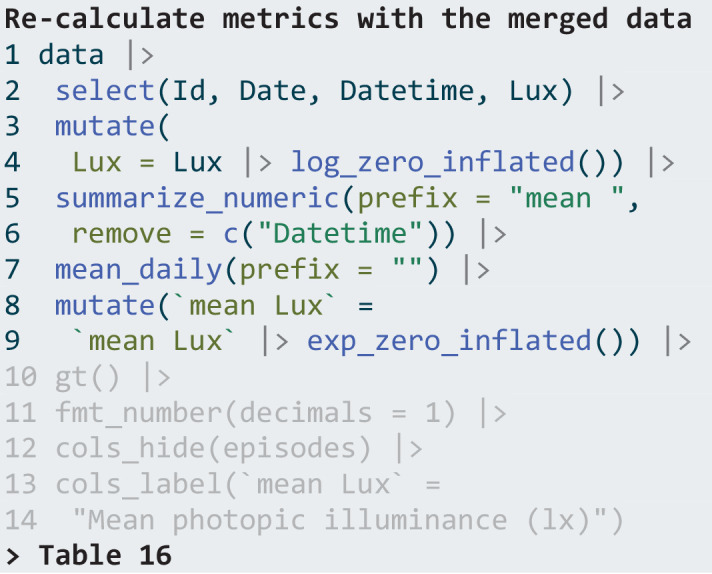

**Line 1** Instead of dataVEET we now supply the merged data object containing data from both devices
**Lines 2–14** Verbatim from Average light exposure

### Spectrum

The VEET device's spectral sensor provides multimodal data beyond simple lux values, but it requires reconstruction of the actual light spectrum from raw sensor counts. We processed the spectral sensor data in order to compute two example spectrum-based metrics. Detailed data import, normalization, and spectral reconstruction steps are given in Supplement 1; here we present the resulting metrics. Briefly, the VEET's spectral sensor recorded counts in nine wavelength bands (roughly 415 to 910 nm), plus a Dark, a Clear, and a flicker detection channel.^1^ After normalizing by sensor gain and applying the calibration matrix, we obtained an estimated spectral irradiance distribution for each 5-minute interval in the recording. With these reconstructed spectra, we can derive novel metrics that consider the spectral content of the light.

Spectrum-based metrics in wearable data are relatively new and less established compared to distance or broadband light metrics. The following examples illustrate potential uses of spectral data in a theoretical sense, which can be adapted as needed for specific research questions.

#### Ratio of short- vs. long-wavelength light

Our first spectral metric is the ratio of short-wavelength light to long-wavelength light, which is relevant, for example, in assessing the blue-light content of exposure. We define “short” wavelengths as 400–500 nm and “long” as 600–700 nm (which are not standardized thresholds and can be freely adjusted). Using the list column of spectra in our dataset, we integrate each spectrum over these ranges (using spectral_integration()) and then compute the ratio short/long for each time interval. We then summarize these ratios per day.


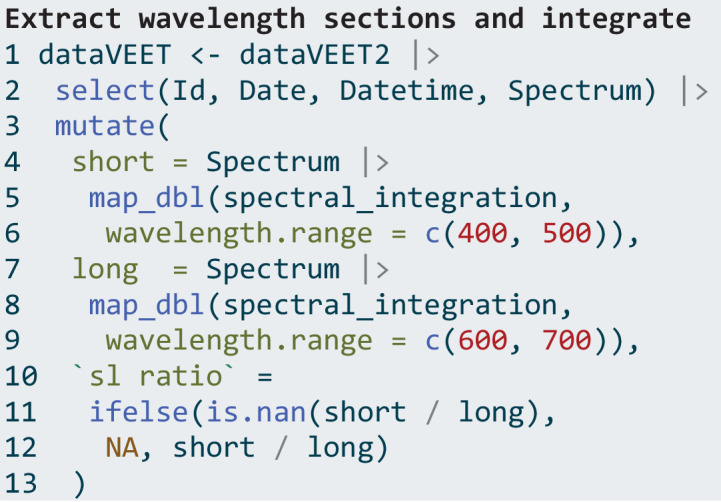

**Line 2** Focus on ID, date, time, and spectrum
**Lines 4–6** Integrate over the spectral range from 400 to 500 nm
**Lines 7–9** Integrate over the spectral range from 600 to 700 nm
**Line 10–12** Compute short-to-long wavelength ratio by using the two integrated values computed on lines 4–9

Table 17 shows the average short/long-wavelength ratio, averaged over each day (and then as weekday/weekend means if applicable). In this dataset, the values give an indication of the spectral balance of the light the individual was exposed to (higher values mean relatively more short-wavelength content).

**Table 17.** Average (mW/m²) and ratio of short-wavelength (400–500 nm) to long-wavelength (600–700 nm) light.

	Irradiance (mW/m²)		
Date	Short	Long	SL-ratio
2025-06-18	83.7	71.7	0.610
2025-06-20	114.0	81.6	0.372


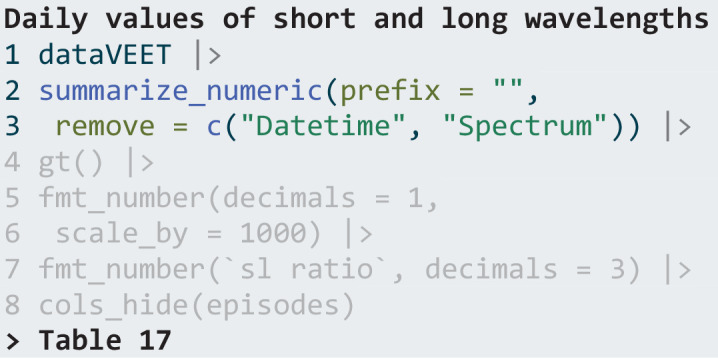


#### Melanopic daylight efficacy ratio

The same idea is behind calculating the melanopic daylight efficacy ratio (or MDER), which is defined by the CIE (2018) as the melanopic equivalent daylight illuminance (melEDI) divided by the photopic illuminance (Hartmeyer & Andersen, 2023). Results are shown in Table 18. In this case, instead of a simple integration over a wavelength band, we apply an action spectrum to the spectral power distribution (SPD), integrate over the weighted SPD, and apply a correction factor. All alphaopic action spectra are implemented in the spectral_integration() function. These will result in photopic illuminance and melEDI.

**Table 18.** Average melanopic daylight efficacy ratio (MDER).

	Illuminance (lx)		
Date	melEDI	Photopic	MDER
2025-06-18	80.6	103.7	0.777
2025-06-20	108.0	123.9	0.871


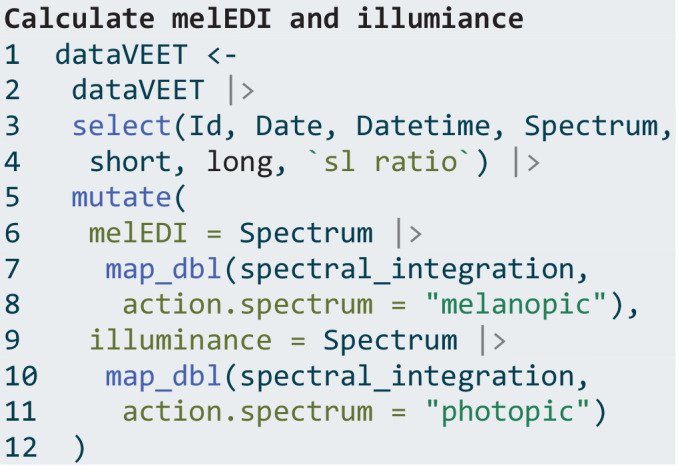

**Lines 6–8** Calculate melanopic EDI by applying the $S_{mel(\lambda )}$ action spectrum, integrating, and weighing
**Lines 9–11** Calculate photopic illuminance by applying the $V_{\left(\lambda \right)}$ action spectrum, integrating, and weighing

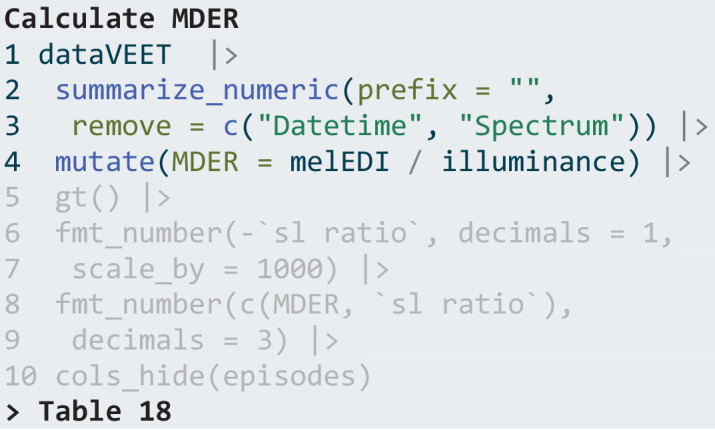


#### Short-wavelength light at specific times of day

The third spectral example examines short-wavelength light exposure as a function of time of day. Certain studies might be interested in, for instance, blue-light exposure during midday versus morning or night. We demonstrate three approaches: (a) filtering the data to a specific local time window and (b) aggregating by hour of day to see a daily profile of short-wavelength exposure. Additionally, we (c) look at differences between day and night periods.

##### Local morning exposure

 Table 19 isolates the time window between 7:00 and 11:00 a.m. each day and computes the average short-wavelength irradiance in that interval. This represents a straightforward query: “How much blue light does the subject get in the morning on average?”

**Table 19.** Average short-wavelength light (400–500 nm) exposure between 7:00 and 11:00 a.m. each day.

Date	Short-wavelength irradiance (mW/m²)
2025-06-18	5.44
2025-06-20	0.95


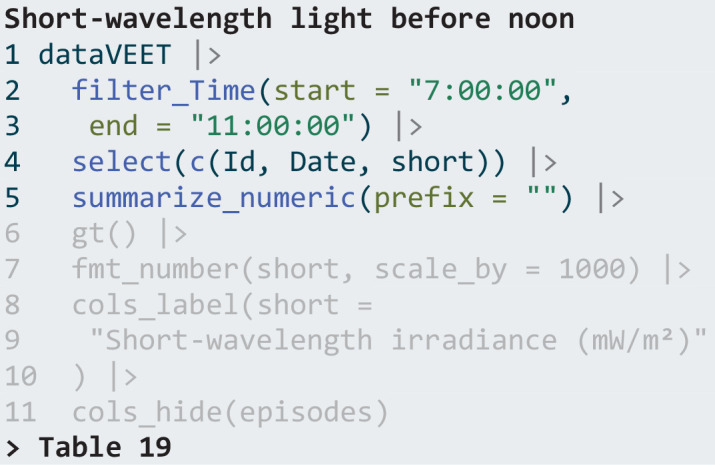

**Lines 2–3** Filter data to local 7 am–11 am

##### Hourly profile across the day

**Figure 11.**
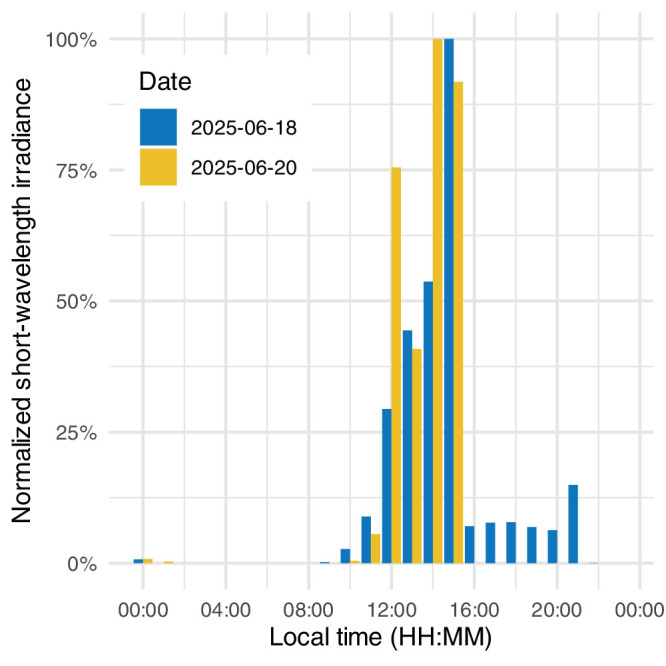
Diurnal profile of short-wavelength light exposure. Each bar represents the average short-wavelength irradiance at that hour of the day (0–23 h), normalized to the daily maximum.


To visualize short-wavelength exposure over the course of a day, we aggregate the data into hourly bins. We cut the timeline into 1-hour segments (using local time) and compute the mean short-wavelength irradiance in each hour for each day. Figure 11 shows the resulting diurnal profile, with short-wavelength exposure expressed as a fraction of the daily maximum for easier comparison.

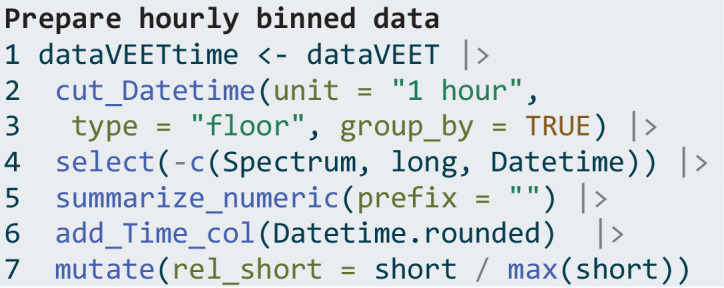

**Lines 2–3** Bin timestamps by hour
**Line 6** Add a time column (hour of day)

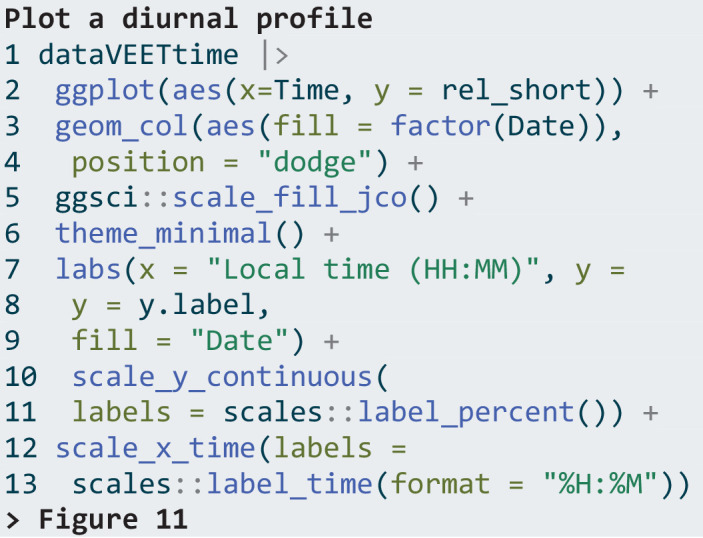

**Line 8**
y.label is a stand-in replacement for “Normalized short-wavelength irradiance”

##### Day versus night (photoperiod)

Finally, we compare short-wavelength exposure during daytime versus nighttime. Using civil dawn and dusk information (based on geographic coordinates, here set for Houston, TX, USA), we label each measurement as day or night and then compute the total short-wavelength exposure in each period. Table 20 summarizes the daily short-wavelength dose received during the day versus during the night.

**Table 20.** Short-wavelength light exposure (mW/m²) during the day and at night.

	Irradiance, mW/m²	
Date	Day	Night
2025-06-18	126.5	12.3
2025-06-20	181.8	1.0


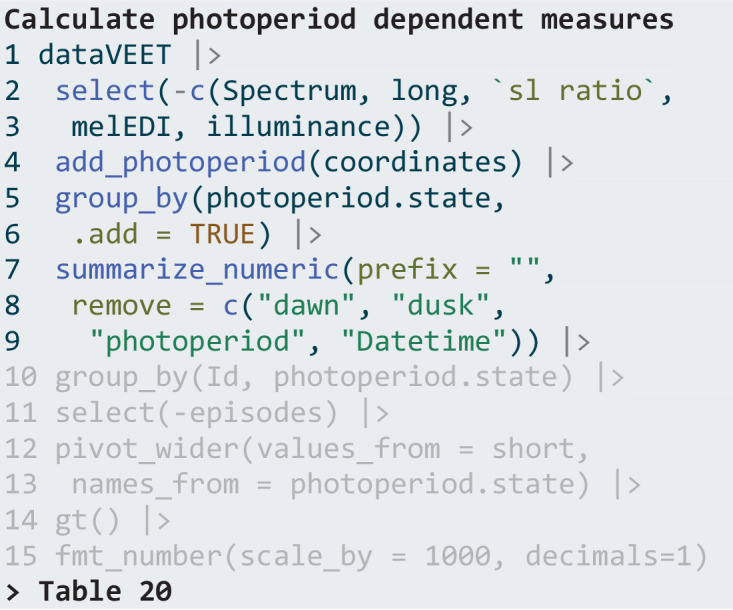


In the code cell above, add_photoperiod(coordinates) is used as a convenient way to add columns to the data frame, indicating for each timestamp whether it was day or night, given the latitude/longitude.

## Discussion and conclusion

This tutorial demonstrates a standardized, step-by-step pipeline to calculate a variety of visual experience metrics. We illustrated how a combination of LightLogR functions and tidyverse workflows can yield clear and reproducible analyses for wearable device data. While the full pipeline is detailed, each metric is computed through a dedicated sequence of well-documented steps, yet remains configurable to realize different metric definitions or thresholds.

By leveraging LightLogR's framework alongside common data analysis approaches, the process remains transparent and relatively easy to follow. The overall goal is to make analysis transparent (with open-source functions), accessible (through thorough documentation, tutorials, and human-readable function naming, all under an MIT license), robust (the package includes >900 unit tests and continuous integration with bug tracking on GitHub), and community-driven (open feature requests and contributions via GitHub).

Even with standardized pipelines, researchers must still make and document many decisions during data cleaning, time-series handling, and metric calculations—especially for complex metrics that involve grouping data in multiple ways (e.g., grouping by distance range as well as by duration for cluster metrics). We have highlighted these decision points in the tutorial (e.g., how to handle irregular intervals, choosing thresholds for “near” distances or “outdoor” light, and deciding on minimum durations for sustained events). Explicitly considering and reporting these choices is important for reproducibility and for comparing results across studies.

As with any behavior-dependent data source, light and distance measurements show substantial interindividual variability, which can lead to marked differences at the individual level. However, the overall processing pipeline generally remains unchanged when applied to group-level analyses. Metric parameters may still require adjustment, particularly when they are determined by device-specific sensor constraints rather than theoretical considerations. For example, the operational definition of near work may vary across populations and devices.

Another important consideration is the role of contextual data in disentangling light and distance patterns into their contributing factors beyond time of day alone. This often requires data sources in addition to the wearable device itself, such as logs or diaries. Zauner and colleagues (2026) describe a strategy for collecting multiple auxiliary data modalities, including activity, sleep, wear time, and other relevant measures. LightLogR provides functions to annotate wearable datasets with such contextual information, enabling more detailed analyses of visual experience data in relation to behavior and environmental context. A free interactive online training course for the package highlights these advanced analytical pipelines and provides specific examples (Zauner & Spitschan, 2025).

The broad set of features in LightLogR—ranging from data import and cleaning tools (for handling time gaps and irregularities) to visualization functions and metric calculators—makes it a powerful toolkit for visual experience research. Our examples spanned circadian-light metrics and myopia-related metrics, demonstrating the versatility of a unified analysis approach. By using community-supported tools and workflows, researchers in vision science, chronobiology, myopia, and related fields can reduce time spent on low-level data wrangling and focus more on interpreting results and advancing scientific understanding.

## Supplementary Material

Supplement 1

## Footnotes

1 Note that older firmware versions of the VEET prior to 2.1.7 contained two Clear channels, and the highest spectral channel was indicated as 940 nm. Data collected with this early firmware version are not suitable for spectral reconstruction in the context of research projects.
